# The Prevalence of Antimicrobial Resistance Genes in the Environments of Small Ruminant Farms from Central Portugal

**DOI:** 10.3390/antibiotics14060576

**Published:** 2025-06-04

**Authors:** Jaqueline T. Bento, Sara Gomes-Gonçalves, Rita Cruz, Fernando Esteves, Alexandra Lameira Baptista, Maria Aires Pereira, Pedro Caseiro, Pedro Carreira, Luís Figueira, João R. Mesquita, Adriano A. Bordalo, Ana Machado

**Affiliations:** 1 School of Medicine and Biomedical Sciences (ICBAS), Universidade do Porto (UP), 4050-313 Porto, Portugal; jtbento@icbas.up.pt (J.T.B.); smggoncalves@icbas.up.pt (S.G.-G.); bordalo@icbas.up.pt (A.A.B.); 2 Escola Superior Agrária de Viseu, Instituto Politécnico de Viseu, Campus Politécnico, 3504-510 Viseu, Portugal; rcruz@esav.ipv.pt (R.C.); festeves@esav.ipv.pt (F.E.); alexabaptista@esav.ipv.pt (A.L.B.); mapereira@esav.ipv.pt (M.A.P.); pedrotenreiro-6@hotmail.com (P.C.); pedropimentacarreira@gmail.com (P.C.); 3 Epidemiology Research Unit (EPIUnit), Instituto de Saúde Pública da Universidade do Porto, 4050-091 Porto, Portugal; 4 Laboratório para a Investigação Integrativa e Translacional em Saúde Populacional (ITR), 4050-600 Porto, Portugal; 5 CERNAS-IPV Research Centre, Instituto Politécnico de Viseu, 3504-510 Viseu, Portugal; 6 Universidade de Trás-os-Montes e Alto Douro, Quinta de Prados, 5000-801 Vila Real, Portugal; 7 Global Health and Tropical Medicine (GHTM), Associate Laboratory in Translation and Innovation Towards Global Health (LA-REAL), Instituto de Higiene e Medicina Tropical (IHMT), Universidade NOVA de Lisboa (UNL), 1349-008 Lisboa, Portugal; 8 Instituto Politécnico de Castelo Branco (IPCB), Av. Pedro Álvares Cabral 12, 6000-084 Castelo Branco, Portugal; lfigueira@ipcb.pt; 9 Associate Laboratory for Animal and Veterinary Science (AL4AnimalS), 1300-477 Lisboa, Portugal; 10 Centro de Estudos de Ciência Animal (CECA), Instituto de Ciências, Tecnologias e Agroambiente (ICETA), Universidade do Porto (UP), 4051-401 Porto, Portugal; 11 CIIMAR-Interdisciplinary Centre of Marine and Environmental Research, University of Porto, 4450-208 Matosinhos, Portugal

**Keywords:** sheep, goat, Portugal, antimicrobial resistance

## Abstract

Background: Antimicrobial resistance is a pressing global concern affecting both human and animal health, with environment playing a key role in the dissemination of resistance determinants. This study aimed to investigate the presence of antimicrobial resistance genes (ARGs) associated with tetracyclines, β-lactams, macrolides, and sulfonamides in environmental matrices collected from 65 sheep and goat farms in central Portugal. Methods: Environmental samples, including water, soil, pasture, and bedding, were analyzed through qPCR for the detection of clinically relevant ARGs. Results: ARGs were detected in 83% of the samples, with over half exhibiting genes from three or more antibiotic classes, suggesting potential multidrug resistance. β-lactamase genes were the most prevalent, followed by those conferring resistance to tetracycline and sulfonamide resistance, while macrolide resistance genes were least frequent. The distribution of ARGs varied by farm type, host species, and municipality. Conclusions: These findings suggest that small ruminant farms serve as important reservoirs for ARGs. The results underscore the need for systematic surveillance and further research into the ecological and genetic factors driving ARG persistence and dissemination in extensive livestock systems, including proper waste management strategies to limit the spread and persistence of antibiotic resistance and mitigate broader public health risks.

## 1. Introduction

Antimicrobial resistance (AMR) refers to the ability of microorganisms to withstand antimicrobial agents, rendering standard treatments ineffective [1]. AMR represents a major global health challenge, demanding coordinated efforts from health authorities across all regions. Within the European Union, it is estimated that over 670,000 infections each year are caused by bacteria resistant to antimicrobial treatments, resulting in approximately 33,000 human deaths annually [1,2]. AMR is recognized as one of the most serious threats to global public health, contributing to driving up healthcare costs, therapeutic failures, and worsened disease outcome or morbidity [3]. The spread of antimicrobial resistance genes (ARGs) among bacterial populations is largely driven by the selective pressure associated with the use and misuse of antimicrobials across multiple domains, including human medicine, veterinary care, and agricultural practices [1]. In response to this complex and multifactorial challenge, the One Health approach has emerged as a comprehensive framework, acknowledging the interconnectedness of human, animal, and environmental health in addressing AMR [4].

Over the years, antibiotics have been administered in food-producing animals not only to treat diseases, but also for non-therapeutic purposes such as improving feed efficiency and growth promotion [5,6]. Following administration, antibiotics are frequently found in the gastrointestinal tracts of livestock at low, sub-lethal concentrations, which inhibit the growth of bacteria that are sensitive to them and confer a selective advantage to those that are resistant [7]. The continuous exposure of animals to low doses of antibiotics plays a major role in the development of AMR, particularly because many of these antibiotics, or their analogs, are also used in human medicine. Long-term studies in veterinary science have consistently demonstrated a clear link between antimicrobial usage and the emergence of resistance [5].

While extensive research has focused on AMR in intensive livestock systems, particularly in swine and poultry industries [8,9], small ruminant farming has received comparatively less attention. In Portugal, sheep and goat farming is a vital component of the agricultural sector, where extensive and semi-extensive systems predominate [10]. Although these systems operate with reduced intensity, they are not exempt from antimicrobial use, which can contribute to the selection and dissemination of ARGs [2,11,12].

The presence of ARGs in livestock environments can pose relevant public health risks, as these genes can be transferred to human pathogens through various pathways, including direct contact, environmental contamination, and the food chain [8]. From a farm production standpoint, antibiotic resistance in livestock negatively impacts both farm production through increased treatment costs and reduced output, and animal fitness (prolonging disease and increasing susceptibility to other health issues). Studies have shown that ARGs are prevalent in livestock waste, soil, and water, facilitating their spread beyond farm boundaries [7,8,13].

Beyond public health implications, the dissemination of ARGs also poses serious ecological risks, reinforcing the relevance of the One Health approach. Antibiotic residues and resistance genes released into the environment, via manure, wastewater, or runoff, can disrupt native microbial communities in soil and aquatic systems, reducing microbial diversity, functional stability, and ecosystem resilience [14]. ARGs can persist in environmental reservoirs, including biofilms and sediments, where horizontal gene transfer (HGT) is enhanced by community interactions and stressors such as heavy metals or pesticides [15]. Collectively, these ecological consequences highlight how environmental dissemination of ARGs can feed back into human and animal systems, reduce the long-term efficacy of antimicrobials, and threaten the ecological functions upon which agriculture and health systems depend.

The antibiotic resistome encompasses the collection of all ARGs present within a particular microbiome, including both pathogenic and non-pathogenic organisms, as well as those not currently expressed [16,17]. This includes AMR organisms such as clinically significant pathogens like *Escherichia coli*, *Klebsiella pneumoniae*, *Pseudomonas aeruginosa*, and *Staphylococcus aureus* [18], which are directly implicated in human and animal infections [19,20]. It also includes commensal and environmental microorganisms that may not cause disease but can serve as reservoirs of ARGs, contributing to their persistence and dissemination [21]. These genes can be mobilized through horizontal gene transfer mechanisms such as conjugation, transformation, and transduction, facilitating the spread of resistance across microbial communities, even in the absence of phenotypic expression [22,23]. This broader perspective acknowledges that resistance genes are a natural component of microbial ecosystems, existing even in environments not directly exposed to anthropogenic antimicrobials [24]. However, the selective pressure exerted by antibiotic use in agriculture and livestock environments can enrich resistant bacteria and mobile genetic elements, increasing the likelihood of horizontal gene transfer [25,26]. As such, studying the resistome provides valuable insights into the potential for resistance emergence and dissemination across environmental, animal, and human reservoirs [27].

The detection of resistance genes associated with four major classes of antibiotics, tetracyclines, β-lactamases, macrolides, and sulfonamides, is often prioritized since these classes are among the most widely used antimicrobials in both veterinary and human medicine, playing a critical role in the treatment of bacterial infections [28,29,30,31]. Tetracyclines and sulfonamides have historically been used extensively in livestock for therapeutic and prophylactic purposes, as well as growth promoters [32,33]. β-lactams, including penicillin and cephalosporins, are commonly used due to their broad-spectrum activity and relatively low toxicity [3,31,34]. Macrolides, although more restricted in veterinary use, are essential for treating respiratory and enteric infections [13,29,30].

As a result, ARGs associated with tetracyclines, β-lactams, macrolides, and sulfonamides are widespread in both clinical and environmental settings, often encoded on mobile genetic elements that facilitate their dissemination [35]. Tetracycline resistance is primarily mediated by efflux pumps and ribosomal protection proteins [36,37]. For β-lactams, resistance typically involves β-lactamase enzymes, which hydrolyze the antibiotic β-lactam ring, rendering it ineffective [3,34]. Macrolide resistance genes include *erm* genes, which encode methyltransferases that modify the 23S rRNA, reducing macrolide binding, and *mef* genes, which code for efflux pumps [38,39]. Sulfonamide resistance is commonly conferred by alternative variants of the dihydropteroate synthase enzyme, encoded by *sul1*, *sul2*, and *sul3*, which have reduced affinity for the drug [40]. The prevalence of these ARGs in various ecosystems underscores the importance of monitoring and controlling their spread to combat AMR.

Despite these findings, data on the occurrence and distribution of ARGs in small ruminant farms in Portugal remain scarce. Understanding the prevalence of these genes is essential for assessing the potential risks associated with small ruminant farming and for developing effective surveillance and mitigation strategies. This study aims to investigate the presence of ARGs in goat and sheep farms located in the central region of Portugal, a major area for the production of such animals. By focusing on this underrepresented sector, the research seeks to contribute to a more comprehensive understanding of AMR dynamics and to support the implementation of One Health-informed policies and practices.

## 2. Material and Methods

### 2.1. Sampling

**Figure 1:**
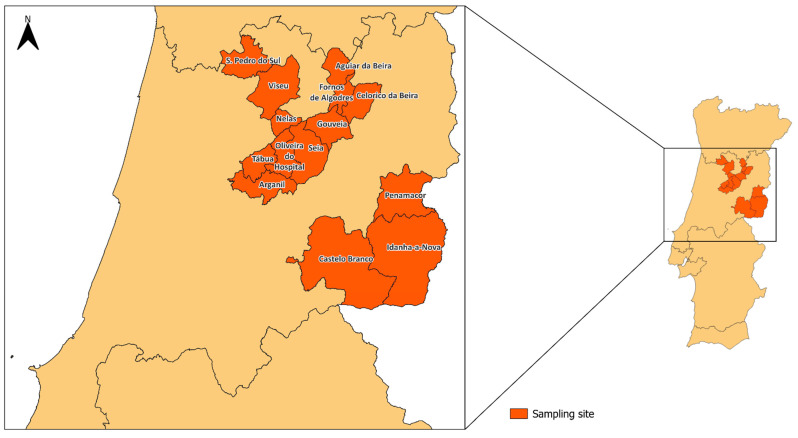
Geographical distribution of samples collected in Portugal. Dark orange represents the municipality from where the samples were retrieved, and the brown yellow the remaining territories of mainland Portugal.

The study was carried out between 29 April 2024 and 25 July 2024 involving environmental samples from 65 different sheep and goat farms. Samples were collected in 14 different locations in the central region of Portugal (Figure 1): Aguiar da Beira (*n* = 8), Arganil (*n* = 3), Castelo Branco (*n* = 1), Celorico da Beira (*n* = 6), Fornos de Algodres (*n* = 1), Gouveia (*n* = 8), Idanha-a-Nova (*n* = 1), Nelas (*n* = 3), Oliveira do Hospital (*n* = 5), Penamacor (*n* = 9), S. Pedro do Sul (*n* = 11), Seia (*n* = 6), Tábua (*n* = 1), and Viseu (*n* = 2) (Table 1). From each farm, the samples collected consisted of water given to animals, farm soil, pasture, and animal bedding. To ensure aseptic sampling and avoid cross-contamination, sterile gloves were worn and changed between each sample and sampling point, and all samples were collected using sterilized equipment directly into sterile containers, which were immediately sealed and transported in insulated boxes with ice packs. Water samples were filtered on-site using sterile membrane filters, used for DNA extraction. All samples were stored and kept at −20 °C until further analysis.

### 2.2. DNA Extraction

Genomic DNA was extracted from 0.25 g of a matrix-mix sample (comprising water, soil, grass, and bedding) [41,42,43] from each farm using the GRS Genomic DNA Kit–Soil^®^ (GRISP, Porto, Portugal), and stored at −20 °C until further analysis.

### 2.3. Molecular Detection of AMR Genes

Molecular detection of AMR genes included β-lactamase genes (*blaCTX-M-9-like*, *blaCTX-M-15-like*, and *bla*TEM), the macrolide resistance gene *ermB*, tetracycline resistance genes (*tetA*, *tetC*, *tetM*, and *tetW*), and sulfonamide resistance genes (*sul1* and *sul2*). For amplification, 0.5 µL of purified DNA was subjected to real-time PCR (qPCR) using 5 µL of iQ^TM^ SYBR^®^ Green Supermix (Bio-Rad, Hercules, CA, USA), and 0.4 µL of each primer (10 µM), in a total reaction volume of 10 µL. For each target gene, positive controls consisted of environmental samples previously confirmed by Sanger sequencing to carry the respective resistance genes, while a no-template consisting in PCR mix and RNAse-free water instead of a DNA template to monitor for potential contamination. Amplification was performed using the following thermal profile: initial denaturation at 95 °C for 10 min, followed by 40 cycles of 95 °C for 10 s, gene-specific annealing temperature (as shown in Table 2) for 30 s, and 72 °C for 30 s. A final extension was carried out at 72 °C for 10 min, followed by a hold at 4 °C. Primer sequences, annealing temperatures, and expected amplicon sizes for each target gene are listed in Table 2. PCR products were separated by 2% agarose gel electrophoresis in 1× TAE buffer (0.04 mol/L Tris-acetate, 0.001 mol/L EDTA [pH 8.0], stained with GreenSafe (Nzytech, Lisbon, Portugal), and visualized under UV light.

### 2.4. Statistical Analysis

The presence or absence of ARGs in each sample was evaluated, and the occurrence was expressed as the proportion of positive samples among the total number of samples analyzed, accompanied by the corresponding 95% confidence interval (95% CI). Statistical differences between groups were assessed using the chi-square and Fisher’s exact test. Data was processed and analyzed using Microsoft Excel^®^ for Microsoft 365 MSO (Redmond, WA, USA) and RStudio version 4.4.2 (Boston, MA, USA).

## 3. Results

To investigate the occurrence and distribution of ARGs in small ruminant farm environments, a total of 65 environmental matrix-mix samples, including water, soil, bedding, and pasture, were collected from sheep (*n* = 45), and goat (*n* = 20) farms located across various municipalities in the central region of Portugal. These samples were screened for a panel of clinically relevant ARGs associated with resistance to key antimicrobial classes.

**Figure 2:**
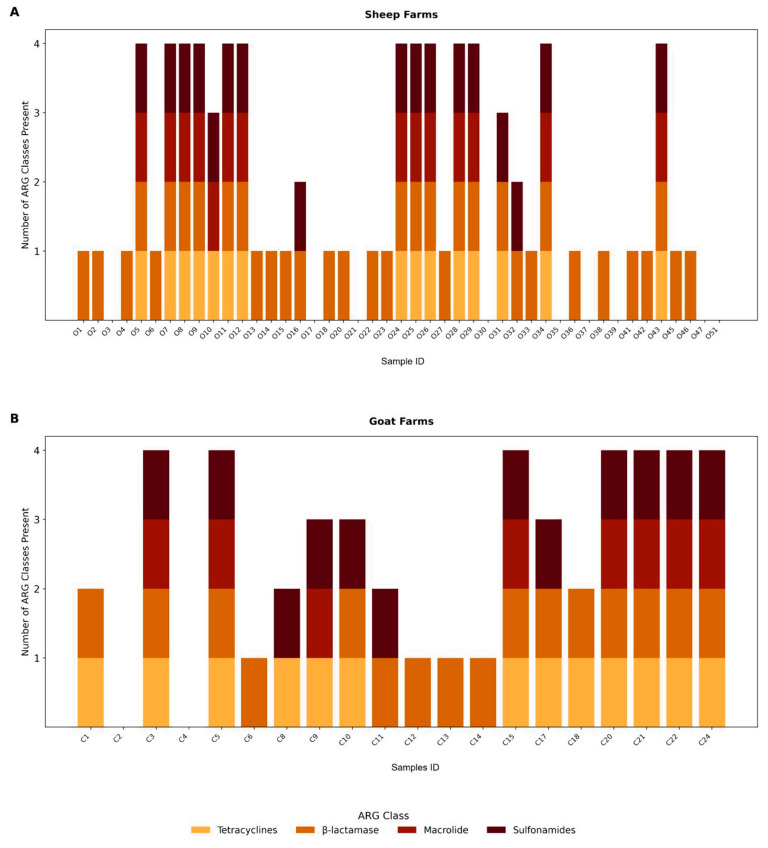
The number of ARG classes detected per individual farm, with sheep farms (*n* = 45) on the upper panel (**A**) and goat farms (*n* = 20) on the lower panel (**B**). Each bar represents a farm, and colors indicate the ARG class: tetracyclines (light orange), β-lactamases (orange), macrolides (red-orange), and sulfonamides (dark red); asterisks represent the absence of ARG. Profiles reflect heterogeneity in ARG class presence across farms and between host species.

ARGs were detected in the majority of the samples, with at least one resistance gene identified in 83.1% of the samples (54/65; 95% CI: 71.7–91.24), indicating widespread environmental dissemination within small ruminant farming systems. Additionally, 55.6% of the samples (25/65; 95% CI: 40.0–70.4) harbored associated with resistance to three or more distinct antimicrobial classes, meeting the criteria for potential multidrug resistance, as illustrated in Figure 2.

**Figure 3:**
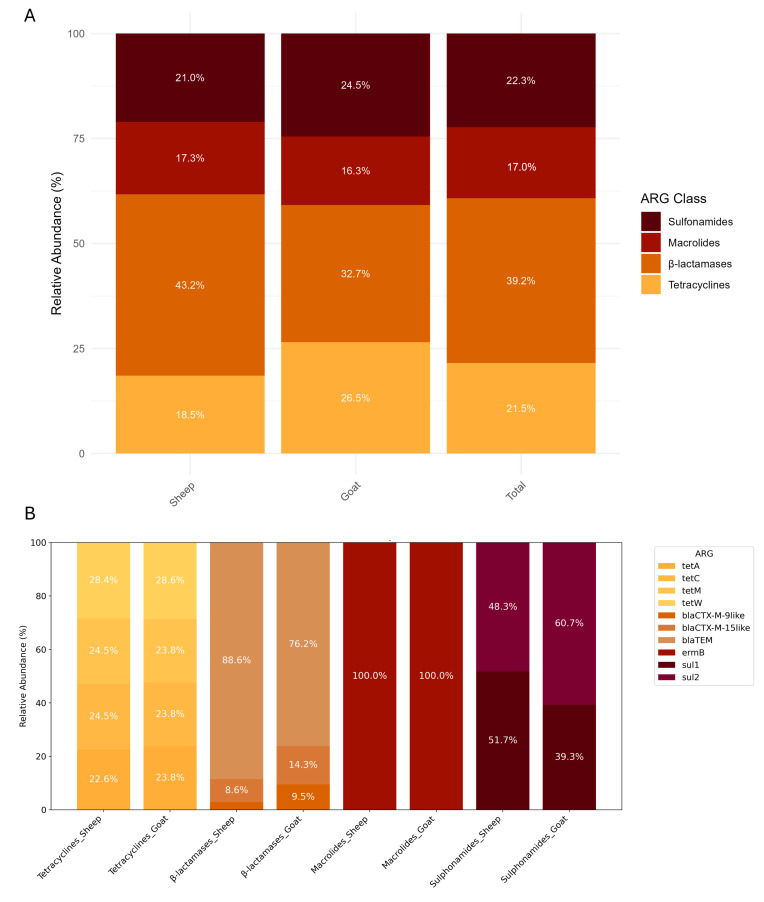
The relative abundance of ARGs by class and gene level. The upper panel (**A**) shows the overall distribution of ARG classes in sheep and goat samples (*n* = 45 and *n* = 20, respectively), as well as the combined total across both hosts, highlighting differences in class dominance between species. The lower panel (**B**) presents the relative contributions of individual ARGs within each class and host species, revealing shifts in gene prevalence across ARG types and host animals.

The distribution of ARGs varied across host species and location. Among all ARG classes, β-lactamase genes were the most frequently detected. These genes were identified in 43.2% of sheep farm samples, and 32.7% of goat farm samples, suggesting a high environmental burden of this resistance mechanism, but without statistical significance (*p* > 0.05). Tetracycline resistance genes were significative more prevalent in goat farms (26.5%) than in sheep farms (18.5%) (*p*-value = 0.035). The remaining ARG classes, including macrolide and sulfonamide resistance genes, showed a more uniform distribution across species and sample types (Figure 3A).

At the gene level, *bla*TEM and *sul1* were more frequently found in samples from sheep farms. In contrast, *blaCTX-M-9-like*, *blaCTX-M-15-like*, and *sul2* were more commonly identified in goat farms. Although tetracycline resistance genes were generally more prevalent in goat environments, their distribution across individual genes, including *tetA, tetC, tetM*, and *tetW*, was balanced between sheep and goat farms (Figure 3B). However, these differences were not statistically significant (*p* > 0.05).

**Figure 4:**
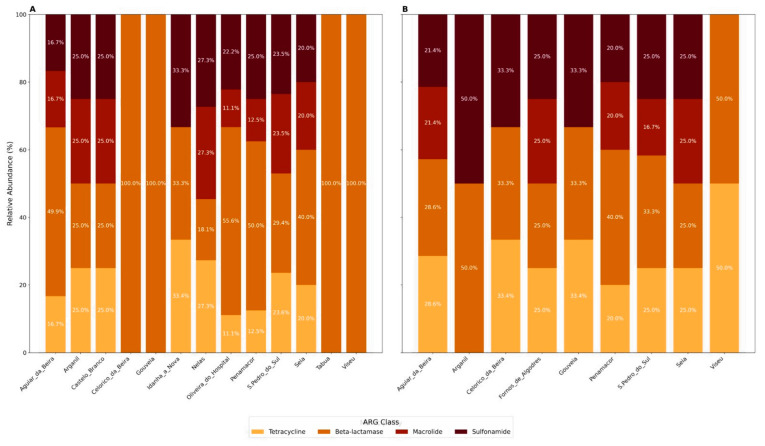
Relative abundances of ARG classes across central Portugal municipalities for sheep (*n* = 13) (**A**) and goat farms (*n* = 9) (**B**). Consistent color coding is used to represent ARG classes, emphasizing variation in resistome profiles by both municipalities and animal hosts.

In terms of ARG class distribution across municipalities (13 for sheep and 9 for goats), β-lactamase genes were detected in all the municipalities surveyed. Sulfonamide and tetracycline resistance genes were more commonly found in municipalities with goat samples (88.8%, 8/9) compared to those with sheep samples (69.2%, 9/13). Macrolide resistance genes were the least frequently observed ARGs, identified in 55.6% (5/9) of goat-sampled and 61.5% (8/13) of sheep-sampled municipalities. Notably, a greater proportion of goat-sampled municipalities (77.7%, 7/9) harbored ARGs from more than three antimicrobial classes, compared to sheep-sampled ones (69.2%, 9/13), as shown in Figure 4. However, Fisher’s exact tests did not reveal statistically significant differences between sheep and goat municipalities for the presence of tetracycline, sulfonamide, macrolide, β-lactamase genes, or for multiresistance (*p* > 0.05 for all comparisons). This suggests that although the relative frequencies differ numerically, these differences are not statistically supported given the current data.

The relationship between the number of ARG classes present, species (goat or sheep), and flock size was examined by fitting a Poisson regression model, using the logarithm of flock size as a predictor, along with species and their interaction. The logarithmic transformation of flock size was applied to normalize its distribution and reduce the influence of extreme values, thereby improving model stability and interpretability. Samples with missing flock size data were excluded from the analysis. Model diagnostics indicated acceptable dispersion (1.180), supporting the robustness of the findings. However, none of the predictors were statistically significant, namely log-transformed flock size (*p* = 0.78), species (*p* = 0.40), and their interaction (*p* = 0.21). Therefore, no significant associations were identified between flock size, species, or their interaction and the number of ARG classes present. Data on flock size are provided in Supplementary Material Tables S1 and S2.

## 4. Discussion

Surveillance of ARGs has gained importance due to the risk of antibiotic-resistant bacteria dissemination, including both zoonotic and non-zoonotic determinants, which pose threats to human and animal health [45]. According to the latest report by the Organization for Economic Co-operation and Development (OECD), Portugal is estimated to have the third highest AMR-related human mortality rate among OECD countries by 2050, underscoring the need of addressing this growing concern [46]. The most recent data from the Institute for Health Metrics and Evaluation (IHME) [47], published in 2021, refer to associated deaths, cases in which a drug-resistant infection contributed to the individual’s death (i.e., the infection was implicated, though resistance may not have been the determining factor) and attributed deaths, which refer to cases where the individual would likely not have died if the infection had been treatable. These estimates focus on six leading drug-resistant pathogens, namely *Staphylococcus aureus*, *Escherichia coli*, *Klebsiella pneumoniae*, *Pseudomonas aeruginosa*, *Acinetobacter baumannii*, and *Streptococcus pneumoniae*.

In Portugal, the estimated numbers of associated and attributed deaths due to antimicrobial-resistant (AMR) organisms were 7008 and 1717, respectively. The leading cause of attributed AMR deaths was methicillin-resistant *Staphylococcus aureus* was the leading cause of attributed AMR deaths in Portugal, with an estimated 764.82 deaths (95% UI: 636.76–892.39), followed by *Escherichia coli* (263.56 [209.92–317.25]), *Klebsiella pneumoniae* (226.95 [191.40–262.50]), *Pseudomonas aeruginosa* (185.07 [151.74–218.41]), *Acinetobacter baumannii* (171.13 [147.71–194.65]), and *Streptococcus pneumoniae* (105.55 [81.78–129.23]).

Increasing evidence suggests that ARGs enter the environment through animal manure, either by direct application or runoff, contaminating soil, water sources, and agricultural crops [48,49]. This can be explained as animals absorb or metabolize only a small portion of antibiotics, excreting through feces or urine approximately 75% of the administered dose [50]. The presence of ARG on small ruminant farm environments remains understudied in Portugal. To address this gap, the current study screened environmental matrix samples for ARGs associated with resistance to key antimicrobial classes, including β-lactamases, tetracyclines, macrolides, and sulfonamides.

The detected widespread presence of ARGs underscores the importance of these farm environments as reservoirs of AMR. With ARGs identified in over 83% of all samples, our findings suggest extensive dissemination of resistance determinants in the environment surrounding sheep and goat farming systems.

Over 60% of the ARGs found were associated with resistance to β-lactams and sulfonamides while the remaining were associated with tetracyclines and macrolides. The high frequency of β-lactamase genes in both sheep and goat farms is of great concern, given that β-lactams are widely used for both prevention and treatment of infections caused by a broad spectrum of Gram-positive, Gram-negative, and anaerobic bacteria [51,52]. The presence of these genes is consistent with previous studies demonstrating the persistence of β-lactamase genes in livestock environments [53,54,55].

Sulfonamides, represented by *sul1* and *sul2* resistance genes, accounted for 22.3% of the total ARGs identified, showing a slightly higher presence across goat farms. Sulfonamides are commonly used to treat a variety of infections, including those affecting the gastrointestinal and respiratory systems in livestock [56]. The persistence of sulfonamides in farm environments is concerning due to their resistance to hydrolysis and stability under acidic pH conditions [57]. Consequently, they are poorly biodegradable, with slow degradation rates in water and soil, resulting in environmental accumulation of residues [57].This resistance could further complicate treatment strategies for both animals and humans, particularly since sulfonamides are also used in human medicine.

While tetracyclines and macrolides were less frequently detected compared to β-lactams and sulfonamides, their presence in the studied farm environments is still noteworthy, as they accounted for approximately 40% of the ARGs found. Tetracyclines are the most widely used veterinary antibiotics, with global usage of tetracyclines in food-producing animals reaching 33,305 tons in 2020 worldwide, with an estimated increase of 9% by 2030 [58,59]. Resistance genes were more prevalent in goat farms samples, aligning with previous reports identifying tetracycline resistance among the most frequently detected in agricultural settings [11,50,60,61]. The high occurrence is likely driven by improper antibiotic use in livestock farming coupled with compound strong affinity for soil particles, which limits biodegradability and prolongs environmental persistence [35,50].

The detection of macrolide resistance genes is consistent with earlier studies in livestock environments [62,63,64], where similar resistance patterns have been observed. This is concerning given the widespread use of macrolides in human medicine, particularly for respiratory and soft tissue infections treatment [65,66]. The continued presence of these genes in agricultural settings raises the potential for cross-resistance between veterinary and human pathogens. The detection of macrolides in these environments raises concerns about the potential for horizontal gene transfer, which could lead to the sharing of resistance mechanisms between bacterial populations in animals and humans [22]. Notably, studies have shown that the concentration of the macrolide resistance gene *ermB* in the gut can increase by 3 to 5 orders of magnitude following antibiotic treatment [22,67].

The co-occurrence of ARGs from three or more antimicrobial classes in over half of the samples analyzed (55.6%) highlights the potential for multidrug resistance (MDR) within these agricultural environments. This trend was particularly evident in goat farms, where 77.7% of the surveyed municipalities exhibited MDR profiles, compared to 69.2% in sheep municipalities. Municipal-level analysis demonstrated that ARGs were widely distributed, with no restriction over specific locations. Notably, β-lactamase genes were detected in all sampled municipalities. Resistance genes conferring sulfonamide and tetracycline resistance were found in over two-thirds of sheep-sampled municipalities, and nearly 90% of goat-sampled municipalities, indicating extensive regional dissemination. Such widespread distribution may suggest that the persistence and spread of ARGs are primarily driven by local agricultural practices, including patterns of antibiotic usage, manure management, and farm hygiene protocols [68,69,70].

The findings presented in this study contribute to the growing evidence that environmental ARGs play a significant role in the broader resistome and may facilitate the transmission of resistance genes to bacteria affecting both humans and animals. This study highlights the need for systematic surveillance of ARGs in livestock farming environments. In addition, a better understanding of the ecological and genetic factors that promote the persistence and dissemination of ARGs in ruminant farms is essential for accurate risk assessment and for designing effective, targeted interventions. As part of those interventions alternatives of antibiotics have been proposed in order to reduce the burden of ARGs dissemination, especially in food-producing animals. Among them are the use of pre- and probiotics as feed additives, fage therapy (bacteriophages), antimicrobial photodynamic therapy, phytochemicals, and vaccines [71,72,73].

Several limitations were encountered in this study. First, the study did not perform extensive quantification of the detected ARGs. Establishing baseline quantitative data for ARG abundance creating a background understanding of their prevalence in these farm environments would enable future studies to more accurately track changes and assess whether ARG abundance increases directly in response to specific factors, particularly the type and intensity of antibiotic administration. This would provide clearer insights into the selective pressures driving ARG proliferation.

It should be noted that a higher number of detected ARGs may indicate an increased copy number of ARGs within individual organisms rather than a greater number of organisms harboring these genes per sample. Since quantitative measurements were beyond the scope of this study, caution is needed when interpreting ARG abundance.

Secondly, the absence of data on the specific antibiotics administered to the herds prevented distinguishing whether detected ARGs stem from natural background levels, as ARGs can be present independent of antibiotic use or pollution [74], or are driven by anthropogenic pressure on antibiotic use. This lack of information prevents a clear distinction between naturally occurring resistance and resistance potentially introduced or amplified by human-related activities. To overcome this limitation, future studies should incorporate records of veterinary antibiotic usage, including types, quantities, and administration schedules, which would enable more precise attribution of resistance patterns to either natural or anthropogenic sources.

Third, while the use of multiple environmental matrices (water, soil, pasture, and bedding) provided a broader overview of the farm ecosystem, it did not allow for precise attribution of ARGs to specific sources. Matrix-specific investigations would help clarify the contribution of each environmental compartment.

Additionally, environmental samples such as soil and water harbor complex microbial communities, which may naturally carry ARGs unrelated to farm activities. Consequently, some detected ARGs may originate from the natural background resistome rather than farm-related contamination. To better distinguish between environmental and farm-associated ARGs, future studies should include comparative analyses with reference sites unaffected by agricultural activity, enabling clearer attribution of ARG sources. Finally, this study did not assess the presence of antibiotic residues in environmental samples. Without residue data, it is hard to determine whether current selective pressures are contributing to the persistence and spread of ARGs. Incorporating the analysis of antibiotic residues alongside ARG detection in future work, would provide a more complete understanding of environmental selection pressures and help clarify the factors driving ARG persistence.

Overall, these findings highlight small ruminant farm environments as important reservoirs of a diverse array of ARGs. Indeed, the observed variability in gene distribution is likely influenced by a combination of antimicrobial usage patterns, animal management practices, and environmental conditions. The presence of multidrug resistance and the co-occurrence of multiple ARG classes underscore the potential for horizontal gene transfer and long-term environmental persistence. Continuous monitoring of ARGs in these ecosystems is essential for assessing potential ecological risks and guiding strategies to mitigate the spread of AMR in agricultural settings.

## 5. Conclusions

This study highlights small ruminant farms in central Portugal as important environmental reservoirs of ARGs, with 83% of the samples testing positive for at least one resistance determinant. The detection of genes associated with β-lactams, sulfonamides, tetracyclines, and macrolides across various environmental matrices and municipalities underscores the widespread and multifactorial nature of ARG dissemination. Notably, over half of the samples exhibited profiles indicative of potential multidrug resistance, reinforcing concerns over the possible horizontal gene transfer and long-term environmental persistence. These findings underscore the urgent need for systematic surveillance strategies, alongside targeted research to further clarify the dynamics of ARG persistence and transmission in extensive farming systems.

## Figures and Tables

**Table 1:** Number of goat and sheep farms sampled at each municipality from the central region of Portugal.

Municipality	Goat Farms (*n*)	Sheep Farms (*n*)	Total
Aguiar da Beira	4	4	8
Arganil	2	1	3
Castelo Branco	-	1	1
Celorico da Beira	1	5	6
Fornos de Algodres	1	-	1
Gouveia	2	6	8
Idanha-a-Nova	-	1	1
Nelas	-	3	3
Oliveira do Hospital	-	5	5
Penamacor	2	7	9
S. Pedro do Sul	6	5	11
Seia	1	5	6
Tábua	-	1	1
Viseu	1	1	2
Total	20	45	65

**Table 2:** Primer sequences, annealing temperatures, and expected amplicon sizes used for the detection of ARGs belonging to different antibiotic classes.

Class	Gene	Sequence (5′ > 3′)	Annealing Temperature (°C)	Product Size (bp)	Reference
Tetracyclines	*tetA*	F:GCTACATCCTGCTTGCCTTC R:CATAGATCGCCGTGAAGAGG	60	210	[13]
	*tetC*	F:TGCAACTCGTAGGACAGGTG R:ACCAGTGACGAAGGCTTGAG	60	139	
	*tetM*	F:ACAGAAAGCTTATTATATAAC R:GGCGTGTCTATGATGTTCAC	51	171	
	*tetW*	F:GAGAGCCTGCTATATGCCAGC R:GGGCGTATCCACAATGTTAAC	60	168	
Sulfonamides	*sul1*	F:TGTCGAACCTTCAAAAGCTG R:TGGACCCAGATCCTTTACAG	60	113	[13]
	*sul2*	F:ATCTGCCAAACTCGTCGTTA R:CAATGTGATCCATGATGTCG	60	89	
Macrolides	*ermB*	F: AGGGTTGCTCTTGCACACTC R: CTGTGGTATGGCGGGTAAGT	58	119	[13]
β-lactamase (ESBL)	*blaCTX-M-15like*	F: GCTGGTGACATGGATGAAAG R: TAGGTTGAGGCTGGGTGAAG	60	87	[44]
	*blaCTX-M-9like*	F: GTTGGTGACGTGGCTCAAAG R: GTTGCGGCTGGGTAAAATAG	60	89	
	*bla*TEM	F: TCTGACAACGATCGGAGGAC R: TGCCGGGAAGCTAGAGTAAG	60	86	

## Data Availability

The original contributions presented in this study are included in the article/Supplementary Materials. Further inquiries can be directed to the corresponding author(s).

## Supplementary Materials

The following supporting information can be downloaded at: https://www.mdpi.com/article/10.3390/antibiotics14060576/s1, Table S1: Sheep Farms Data; Table S2: Goat farms Data.
